# Diet in Enteric Fever

**Published:** 1892-09-10

**Authors:** 


### The Practitioner's Mirror and Retrospect.

[*The Editor will be glad to receive offers of co-operation and contributions from members of the profession. All letters should be addressed to* The Editor, The Lodge, Porchester Square, London, W.]

### MEDICINE.

### DIET IN ENTERIC FEVER.

Important as is the question of diet in the treatment of most diseases there are not many in which it is of more importance than in enteric fever, and in which more care should be exercised in regard both to the quality and the quantity of the food ordered. The lesions here, it must be remembered, are in the intestines, and so are exposed to the direct contact of food; therefore that food should be as little irritating as possible, and no more should be given than the patient can assimilate, for anything beyond that must further inflame the already inflamed and ulcerated intestinal tract.

As regards the *quality* of the food, it should, we know, certainly be bland, nourishing, and of such a nature as to be absorbed almost entirely by the stomach and duodenum, leaving little or nothing to be dealt with by the lower part of the small intestine. *Milk* is no doubt the best food where it suits the patient. It is generally advisable that it should be boiled, and diluted with soda-water or lime-water; but where there is any tendency to constipation, barley-water may be substituted. By mixing one of these with the milk the casein curdles in a state of more minute subdivision, and so is made more easy of digestion. Should milk still disagree, try peptonizing it; and if then undigested curd be found in the stools give whey alone, in which case Dr. Wallace Beatty recommends that beef-tea with the grounds, or beef juice should be given along with the whey to take the place of the albuminate casein. Milk, then, should be the staple food, but other nourishment may generally be allowed, such as mutton and chicken broths, beef-tea, or calves'-foot jelly." Beef-tea has often been objected to, for it is said that it causes tympanites and increases the diarrhœa; and speaking of this latter point, Fagge says: "Experience fully bears this out," but adds, "it must, however, be remembered that in many cases of enterica there is no diarrhœa, but constipation throughout the fever. Then beef-tea is indicated rather than not, and its valuable effect on the heart should not be lost." In cases where milk cannot be taken, or not in sufficient quantity, fresh eggs may be given, raw and well beaten up; and Brand's essence of beef is generally readily assimilated.

Now, as regards the *quantity* of food to be given. Graves in his day was almost alone in giving a proper amount of nourishment to fever patients. In his *Clinical Medicine* he says, "Many physicians supposed fever arose from inflammation, and concluded that to treat it successfully it was necessary to reduce the system by depletion and low diet . . . If a patient's face was flushed or his eyes suffused . . . they said, 'Here is inflammation of the brain, and nourishment will exasperate it.' If he had red or dry tongue or abdominal tenderness, they instantly inferred the existence of gastro-enteritis, and all kinds of food, even the lightest, were strictly forbidden." Graves protested against this starvation, and felt so earnestly about it that, as is well known, he wished to have engraved on his tomb the epitaph, "He fed fevers." If he were alive now his efforts would have to be exerted in the other direction, for the profession has rushed into the opposite extreme, and the stomachs of fever patients are often most distressingly overloaded in the endeavour to "support them under the ravages of the disease." Dr. Broadbent* says, "The attention of the medical attendant will in most cases be required rather to moderate the amount of food given than to urge its administration. The key to the regulation of the diet . . . is to be found in the careful inspection of the stools. . . When curds appear in the dejections, either too much milk is taken, or too much at a time, or its digestion is interfered with." He allows altogether about 4½ pints of liquid food; but Dr. Wallace Beatty thinks this amount excessive, and would limit it to two or three pints in the twenty-four hours. In an article in the *Dublin Journal of Medical Science** he is of opinion that a more restricted diet than most physicians allow would tend to avert, or, at least, to mitigate the severity of several distressing symptoms and complications, such as diarrhœa, hæmorrhage, tympanites, sleeplessness, and delirium. Sir William Jenner and Fagge would both allow the patient to drink as much pure water as he wished, but Beatty would limit the amount even of this, and would seek to quench the thirst by means of ice, or lemonade, or a little tea. So we may conclude that three or at the outside four pints of liquid food in the twenty-four hours is sufficient nourishment for an adult, and that proportionately less should be given to children.

When the patient becomes convalescent, great caution must be exercised in the alteration of the food; so many relapses have apparently started from some imprudence in diet. Brigade-Surgeon A. A. Gore† tells of a case in which "twelve days after normal temperature, a severe relapse with high temperature (104·8 deg.), quickened pulse, and diarrhœa followed upon the first use of chicken diet." In another case a child was recovering from a very mild attack of typhoid fever, she had no spots or diarrhœa, and her temperature never rose above 101 deg. She was given a little bread and butter, and the next day she had a relapse; her temperature rose, spots appeared, typical stools were passed, then followed hæmorrhage, abdominal pain, delirium, and death. And many other such cases are on record. Fagge says that no solid food should be given for a fortnight after the fever and diarrhœa have ceased. Beatty, in the article already quoted, says: "Convalescence from enteric fever may be regarded as begun if there are no spots present, if the temperature has been normal for at least a week, if the tongue is moist, if the abdomen is not tympanitic, if the spleen is not enlarged, and if the stools are formed and of a natural colour." He gives the following directions about the way in which the change of food should be made: "The safest way is to increase very gradually the consistence of the food. For a few days, indeed, it is safer to increase the quantity of the food which has been given during the illness, rather than to make any change in its nature. I change the diet much as follows: First I give milk thickened with cornflour or arrowroot, then after a few days a lightly-boiled egg and a few plain biscuits, then bread, then fish, and so on; all along I watch the temperature carefully, so as to detect any rise at once."

As regards the administration of alcohol in typhoid fever, very much depends upon the age of the patient and the course of the disease. If the patient be young, or if the fever be mild and run a straightforward course, probably none will be needed. The heart should be carefully watched, and if the first sound becomes faint and weak, it should be withheld no longer. It will be necessary to administer it also if there be much prostration, or if the patient be old and feeble, and in these cases it should be given freely, but always in definite doses. Brandy is a very suitable form, but sometimes champagne suits old people better.

* "Typhoid Fever."—*Quain's Dict. of Med.*

* "The Dietetic Treatment of Enteric Fever."—May 21, 1892.

† "Analysis of 105 Cases of Enteric Fever." *Dublin Journal*, August, 1891.

